# Th17 Polarization under Hypoxia Results in Increased IL-10 Production in a Pathogen-Independent Manner

**DOI:** 10.3389/fimmu.2017.00698

**Published:** 2017-06-19

**Authors:** Roman Volchenkov, Vegard Nygaard, Zeynep Sener, Bjørn Steen Skålhegg

**Affiliations:** ^1^Department of Nutrition, Institute of Basic Medical Sciences, University of Oslo, Oslo, Norway; ^2^Department of Core Facilities, Institute for Cancer Research, Oslo University Hospital HF – Radiumhospitalet, Montebello, Oslo, Norway

**Keywords:** Th17 cells, IL-17, oxygen, cigarette smoke, aryl hydrocarbon receptor

## Abstract

The IL-17-producing CD4^+^ T helper cell (Th17) differentiation is affected by stimulation of the aryl hydrocarbon receptor (AhR) pathway and by hypoxia-inducible factor 1 alpha (HIF-1α). In some cases, Th17 become non-pathogenic and produce IL-10. However, the initiating events triggering this phenotype are yet to be fully understood. Here, we show that such cells may be differentiated at low oxygen and regardless of AhR ligand treatment such as cigarette smoke extract. Hypoxia led to marked alterations of the transcriptome of IL-10-producing Th17 cells affecting genes involved in metabolic, anti-apoptotic, cell cycle, and T cell functional pathways. Moreover, we show that oxygen regulates the expression of CD52, which is a cell surface protein that has been shown to suppress the activation of other T cells upon release. Taken together, these findings suggest a novel ability for Th17 cells to regulate immune responses *in vivo* in an oxygen-dependent fashion.

## Introduction

IL-17-producing CD4^+^ T helper cells (Th17) are important for pathogen clearance and tissue inflammation and are considered as a hallmark cell in a number of autoimmune diseases including rheumatoid arthritis (RA) (1). Their development is dependent on activation of hypoxia-inducible factor 1 alpha (HIF-1α) (2), and they are polarized by cytokines like TGF-β, IL-23, IL-6, and IL-1β (1). Furthermore, stimulation of the transcription factor (TF), RORγt through the aryl hydrocarbon receptor (AhR) pathway by a number of ligands including miscellaneous metabolites and environmental factors such as cigarette smoke (CS), affects the activity of Th17 cells (3, 4). There is a growing body of evidence that Th17 cells might be subdivided into pathogenic and non-pathogenic (5, 6) lineages. While pathogenic Th17 cells are extensively studied, the non-pathogenic subsets are poorly understood. It is believed to be pathogen and IL-1β dependent (6), and possibly regulated by the proto-oncogene c-MAF that belongs to the MAF family of TFs (7).

Low oxygen environment or hypoxia is a normal state of many tissues and compartments in the human body (8). Moreover, it has been demonstrated that for various types of cells, including embryonic stem cells, hypoxia is a necessary condition for proper differentiation (9, 10). However, most of the studies nowadays are still performed at atmospheric O_2_ concentrations, which are considerably higher of those in peripheral blood and peripheral tissues (8). Immune cells, and T cells in particular, are not an exception, and their development and function are highly dependent on oxygen conditions (11). Under hypoxia, most of the endogenous changes and hence metabolic and functional activities are attributed to expression and accumulation HIF-1α. HIF-1α is also upregulated in naïve CD4^+^ T cells by several unspecific ligands including lipopolysaccharide and specific ligands such as antigen stimulation of the T cell receptor (TCR) complex. Moreover, several pro-inflammatory cytokines such as IL-1β, IL-6, and TNF-α also induce HIF-1α expression. In addition, HIF-1α can be induced by nutrient-stimulated mTORC1 activation followed by activation of genes supporting metabolic activity including glycolysis. In either case, upregulation of HIF-1α supports differentiation of Th17 cells through the master regulator RORγt, which together with the protein p300 induce optimal transcription at gene loci associated with the Th17 phenotype (2). It is also demonstrated that HIF-1α binds to and mediates proteasomal degradation of Foxp3, preventing differentiation of T regulatory (Treg) cells (2). Together this suggests that HIF-1α drives expression of survival genes that aid in Th17 persistence and is expected to prevent Treg-dependent tolerance (8).

In this study, we have shown that a low oxygen atmosphere but not constant exposure to AhR ligands such as cigarette smoke extract (CSE) results in differentiation of Th17 into a non-pathogenic state, characterized by increased secretion of IL-10 despite exposure to the standard polarizing cytokines used for Th17 *in vitro* differentiation and without addition of specific pathogen.

## Materials and Methods

### Blood Samples, Cell Differentiation, and Treatment

Buffy coats from anonymous healthy blood donors were prepared at Oslo Bloodbank (Ullevål hospital). Informed consent from all subjects was obtained prior to donation by the Oslo Bloodbank according to the Norwegian laws and regulations. All donors CD4^+^ T cells from buffy coats were isolated by positive selection using magnetic beads (Dynal/Fisher). Flow cytometry confirmed purity as 96% (CD3^+^CD4^+^ staining).

T cells were cultured in RPMI 1640 medium with 10% heat inactivated calf serum, 1% penicillin/streptavidin, and 2% l-glutamine. Cells were plated in 12 well plates (0.2 × 10^6^ per well) and stimulated by anti-CD3/CD28 beads (Dynal/Fisher) with 1:2 bead-to-cell ratio for 7 days in atmosphere of 5% CO_2_ or 5% CO_2_ and 1% O_2_. Th17 populations were polarized per protocol from Miltenyi Biotec by addition of recombinant IL-1β (20 ng/mL, BD), IL-6 (30 ng/mL, BD), IL-23 (30 ng/mL, R&D systems), TGF-β (2.25 ng/mL, BD), and neutralizing antibodies to anti-IFN-γ (1 µg/mL, clone B27, BD) and anti-IL-4 (2.5 µg/mL, clone MP4-25D, BD). CSE (40 mg/mL) was prepared by Murty Pharmaceuticals by smoking of University of Kentucky’s 3R4F Standard Research Cigarettes on an FTC Smoke Machine and subsequent extraction from the filter. To measure the proliferation, the T cells were labeled with Cell Trace Violet per manufacturer’s protocol (Life Technologies). Cell death was measured using either propidium iodide or trypan blue staining (Sigma). Cell counting was done using a Countess cell counter (Invitrogen).

Electron transport chain (ETC) experiments were performed by addition of rotenone, antimycin A, NaCN, and CCCP uncoupler to standard Th17 polarization cocktail both under 21% O_2_ and 1% O_2_.

### Flow Cytometry

T cells were labeled with monoclonal antibodies against CD4 (OKT4), CD25 (BC96), CD38 (HIT1), CD48 (TU145), CD52 (4C8), CD127 (HIL-7R-M21), CD161 (HP-3G10), CD196 (11A9), and CD226 (DX11), all from BD or Biolegend, as described previously (12). Intracellular staining for HIF-1a (241812 from R&D systems) and AhR (T49-550 from BD) was done after fixation and permeabilization using kit from R&D Systems. The profiling was done after 7 days in culture if not mentioned otherwise in the figure legend. The data acquisition was done at LSRFortessa (BD biosciences), and analysis was performed with FlowJo v10 software (Tree star).

### Cytokine Analysis

Secretion of cytokines was measured in supernatants from T cell cultures after 7 days (if not mentioned otherwise in the figure legend) using magnetic beads based methods—Legendplex (IL-17A, Biolegend) or Bio-Plex (TNF-α, IL-6, IL-10, IFN-γ, IL-4, IL-2; Bio-rad). To test if CSE causes block of cytokine secretion, the cells were fixed permeabilized using Cytofix/Cytoperm (BD biosciences) per manufacturer’s protocol and stained with anti-IL-17A (clone N49-653 from BD).

### Bioenergetic Measurements

Oxygen consumption rates (OCRs) of CD4^+^ T cells were measured in non-buffered RPMI 1640 medium supplemented with 10 mM glucose, 2 mM l-glutamine, and 1 mM sodium pyruvate under basal conditions and in response to 1 µM oligomycin, 1.5 µM carbonyl cyanide m-chlorophenyl hydrazone (CCCP), and 100 nM rotenone + 1 µM antimycin A (Sigma). Extracellular acidification rate was measured in non-buffered RPMI 1640 containing 2 mM l-glutamine and 32 mM additional sodium chloride under basal conditions in response to 10 mM glucose, 1 µM oligomycin, and 20 mM 2-DG with Extracellular XF24e Flux Analyzer (Seahorse Bioscience).

### Transcription Profiling and Pathway Analysis

T cells were collected after 7 days in culture, and total RNA was extracted using QIAgen RNeasy kit per manufacturer’s instructions. RNA amplification, labeling, and hybridization to IlluminaHT-12 v4 Human expression array were performed at Oslo University Hospital Genomics Core Facility. The GenomeStudio software from Illumina was used to summarize the signals per gene and perform quintile normalization and imputation for missing probes (“Sample Gene Profile”). Linear Models for Microarray and RNA-seq Data (LIMMA) software package was used with log 2-transformed data to find differentially expressed genes (DEGs) (13). Hierarchical clustering with Euclidean distance and complete linkage was performed by the function heatmap.2 within the R-package gplots. Genes with *p* values lower than 0.005 were used for subsequent pathway analysis using Ingenuity Pathway Analysis (IPA) software (QIAgen). Functionally grouped networks were visualized using Cytoscape v3.5.1 software with GlueGo plug-in with up to date Gene Ontologies (GO, Gene Ontology Consortium, http://geneontology.org) (14). The script used to analyze the microarray data and make the related figures and tables is available at https://github.com/ous-uio-bioinfo-core/volchenkov-et-al-2016.

### Statistics

Statistical analysis was performed using GraphPad Prism 5. Two-way ANOVA was used for proliferation, cytokines, bioenergetics measurements, and surface marker expression. Significance was set at *p* < 0.05.

## Results

### Treatment with CSE Suppresses Th17 Differentiation in a Dose-Dependent Manner

Environmental factors, including high salt diet (15) and microbiome (16), have been proposed to have an effect on the development of autoimmunity but so far, cigarette smoking is the only environmental factor linked to the risk of RA initiation and development (17). Components of CS and CSE contain a large number of chemicals, many of which have cancerogenic properties (18) and trigger AhR signaling in the cells. Although AhR stimulation is directly involved in the development of Th17 and CS has been shown to increase IL-17A and IL-17F expression in lungs, little is known about the direct effect of CS components on the development of Th17 cells (3, 19). To address this, we tested the effect of CSE on Th17 cell polarization upon stimulation of the TCR/CD3 complex and in the presence of standard cytokines used for Th17 differentiation (20).

**Figure 1:**
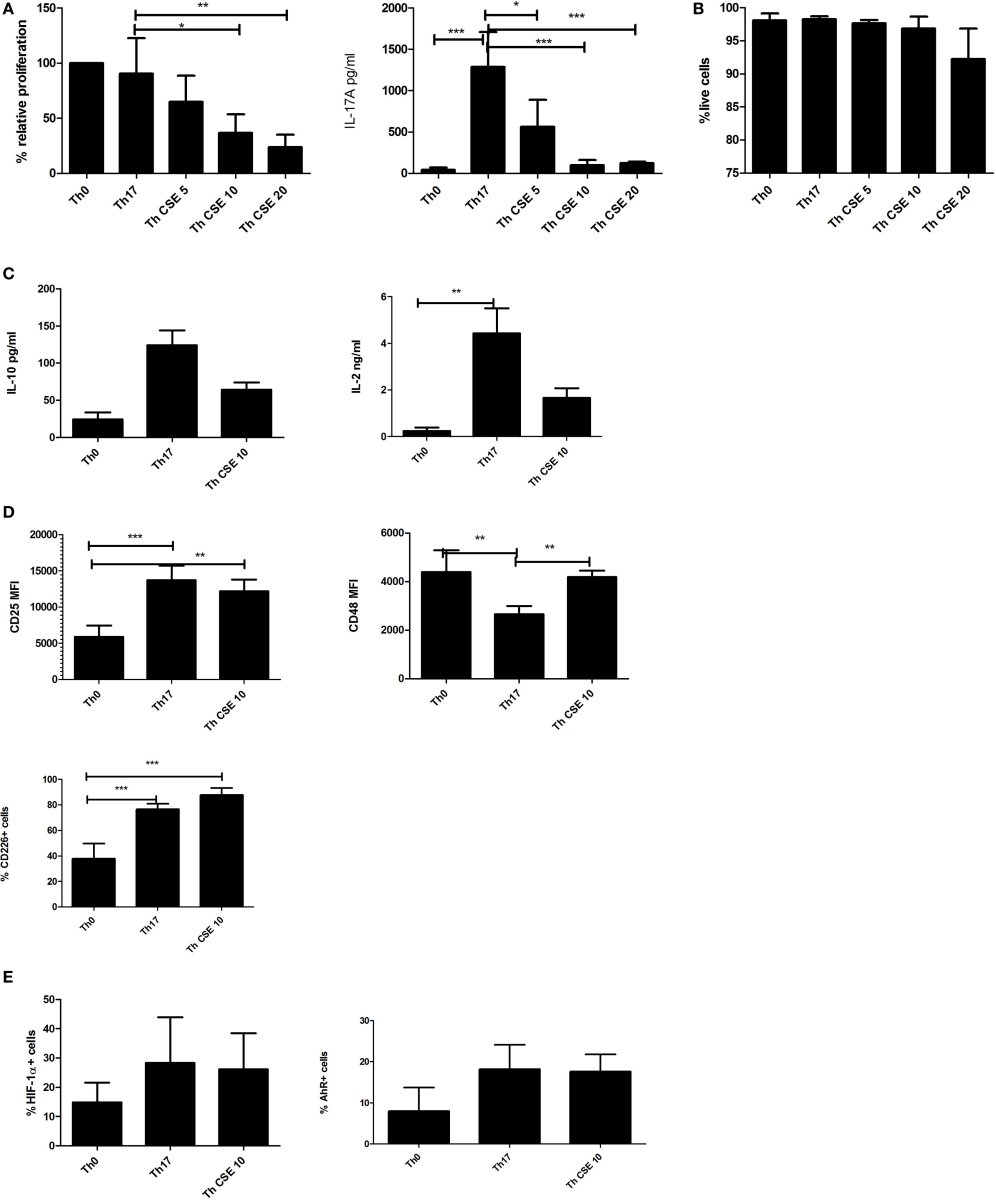
Cigarette smoke extract (CSE) affects Th17 proliferation and IL-17 production in a dose-dependent manner. T cells were stimulated by anti-CD3/CD28 beads (Th0) and polarized in presence of Th17 cocktail (Th17) with addition of CSE (Th CSE). **(A)** CSE reduces proliferation of T cells in a dose-dependent manner and IL-17A production by Th17 cells. **(B)** CSE treatment was not associated with reduced viability. **(C)** CSE treatment has mild effects on other cytokines. **(D)** CSE restores CD48 and increases CD25 and CD226 expression on the cell surface. **(E)** Both Th17 and Th CSE increase HIF-1α and aryl hydrocarbon receptor (AhR) levels, although insignificantly. The bars show mean + SD, *n* ≥ 3 in all experiments (*p < 0.05; **p < 0.005; ***p < 0.001).

After 7 days in culture CSE treatment (0–20 µg/mL) was associated with reduced proliferation (Figure 1A, left panel) and significantly affected the IL-17A production in a dose-dependent manner (Figure 1A, right panel). The reduction in proliferation was not associated with cell death (Figure 1B). To eliminate the possibility that CSE prevented secretion of IL-17A, we stained for intracellular IL-17A without a Golgi block demonstrating that no endogenous IL-17A was detectable (see Figure S1 in Supplementary Material). We next investigated if the reduction in IL-17A was associated with altered levels of TNF-α, IL-10, and IL-2. This showed that TNF-α levels were unaffected (data not shown), whereas for IL-10 the differences were non-significant (Figure 1C, left panel). The IL-2 production was significantly higher in Th17 cells vs Th0 (unpolarized yet TCR-stimulated cells) (Figure 1C, right panel).

Next, we investigated the expression of cell surface proteins related to activation of T cells such as CD25, CD48, and CD226 (21, 22). Expression of these receptors was significantly altered between Th0 cells, Th17, and Th CSE (Figure 1D). For instance, Th17 polarization was associated with a significant upregulation of CD25, which was unaltered by CSE (Figure 1D, upper left panel). In the case of CD48, Th17 polarization was associated with a significant reduction, which was abrogated by CSE exposure (Figure 1D, right panel). Finally, both Th17 and Th CSE cells showed significantly increased expression of CD226 (Figure 1D, lower left panel). As HIF-1α expression and AhR stimulation are both involved in Th17 polarization, endogenous expression of these two proteins was examined and we found them to be non-significantly upregulated by Th17 polarization and unaffected by CSE (Figure 1E).

### CSE Treatment Affects the Transcriptional Profile of Th17 Cells by Preventing Effects of Polarization

Our data showed that Th17 proliferation and IL-17A production and hence Th17 polarization were affected by CSE, at the same time neither HIF-1α nor AhR expression were dramatically altered. To further understand this T cell phenotype driven by CSE further, we investigated the transcriptional changes by employing the Illumina HT-12 v4 Human expression array.

**Figure 2:**
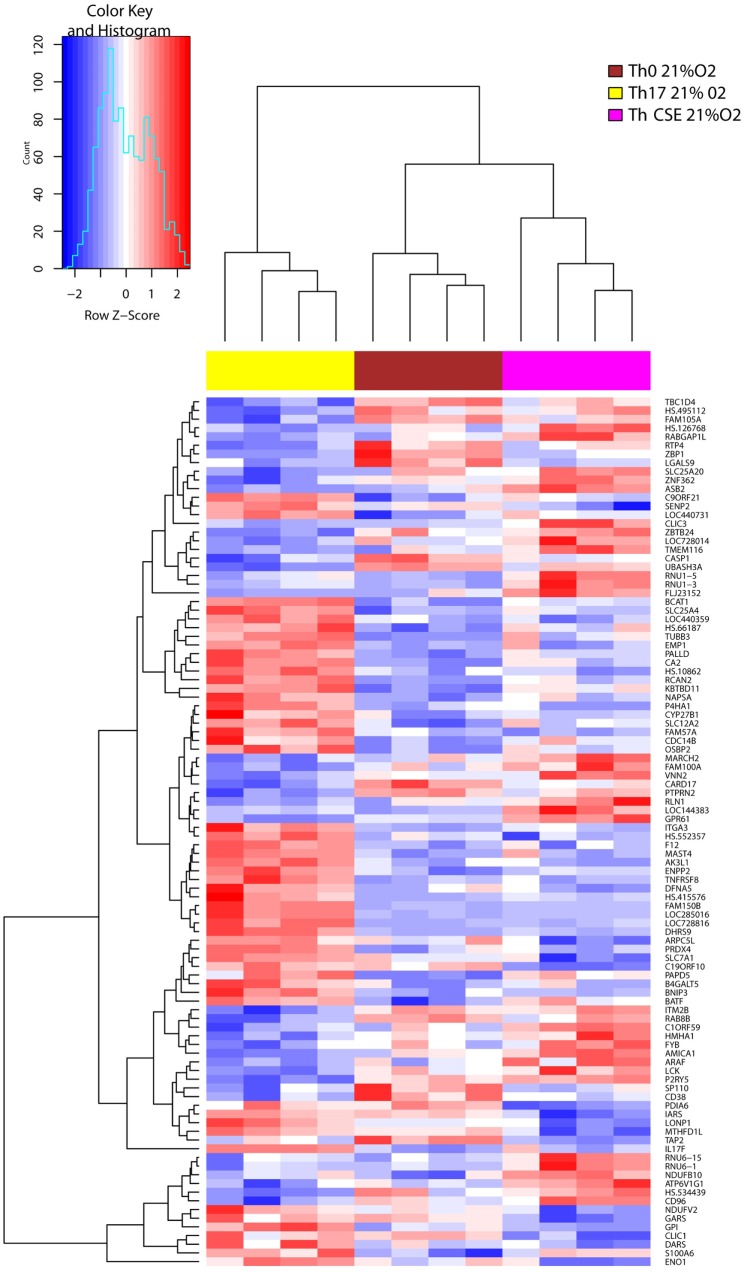
Cigarette smoke extract (CSE) treatment removes the effects of Th17 polarization. Two-way hierarchical clustering of 108 differentially expressed genes observed in three T cell populations (Th17, Th CSE, and Th0, *n* = 4) at 21% O_2_. Th CSE appears similar to Th0.

Hierarchical clustering of change in the gene expression patterns of Th0, Th17, and Th CSE cells is shown in Figure 2. Here, we only observed six DEGs between Th CSE and Th0 cells. These included upregulation of mRNA for *NDUFB10* (Complex I of the ETC); as well as the small snRNAs *RNU1–5* and *RNU1–3*, which aid in the regulation of TFs (7SK RNA) or RNA polymerase II (B2 RNA), as well as maintaining the telomeres (23). Finally, *LGALS9* (lectin, galectin-9), *PDIA6* (protein disulfide isomerase), and *TAP2* (immune cell-related multidrug resistant membrane transporter) were also downregulated.

In line with the cytokine profile, Th17 cells upregulated signature gene *IL17F* in comparison to control cells (Th0), and this effect was diminished in Th CSE cells further supporting experimental evidence that lack of IL-17 in the supernatant was not due to block of secretion of the cytokine from cells treated with CSE (Figure 2). CSE treatment also reduced levels of mRNA for the mitochondrial protein synthesis enzymes aspartyl-, glycyl-, isoleucyl-tRNA synthetases, *DARS, GARS*, and *IARS* (24). In addition, we also observed reduced levels of mRNA for glucose-6-phosphate isomerase (*GPI*) and enolase 1 (*ENO1*), which are enzymes involved in glycolysis and gluconeogenesis.

### Hypoxia Affects the Cytokine Profile of T Cells and Shapes the Th17 Cells

**Figure 3:**
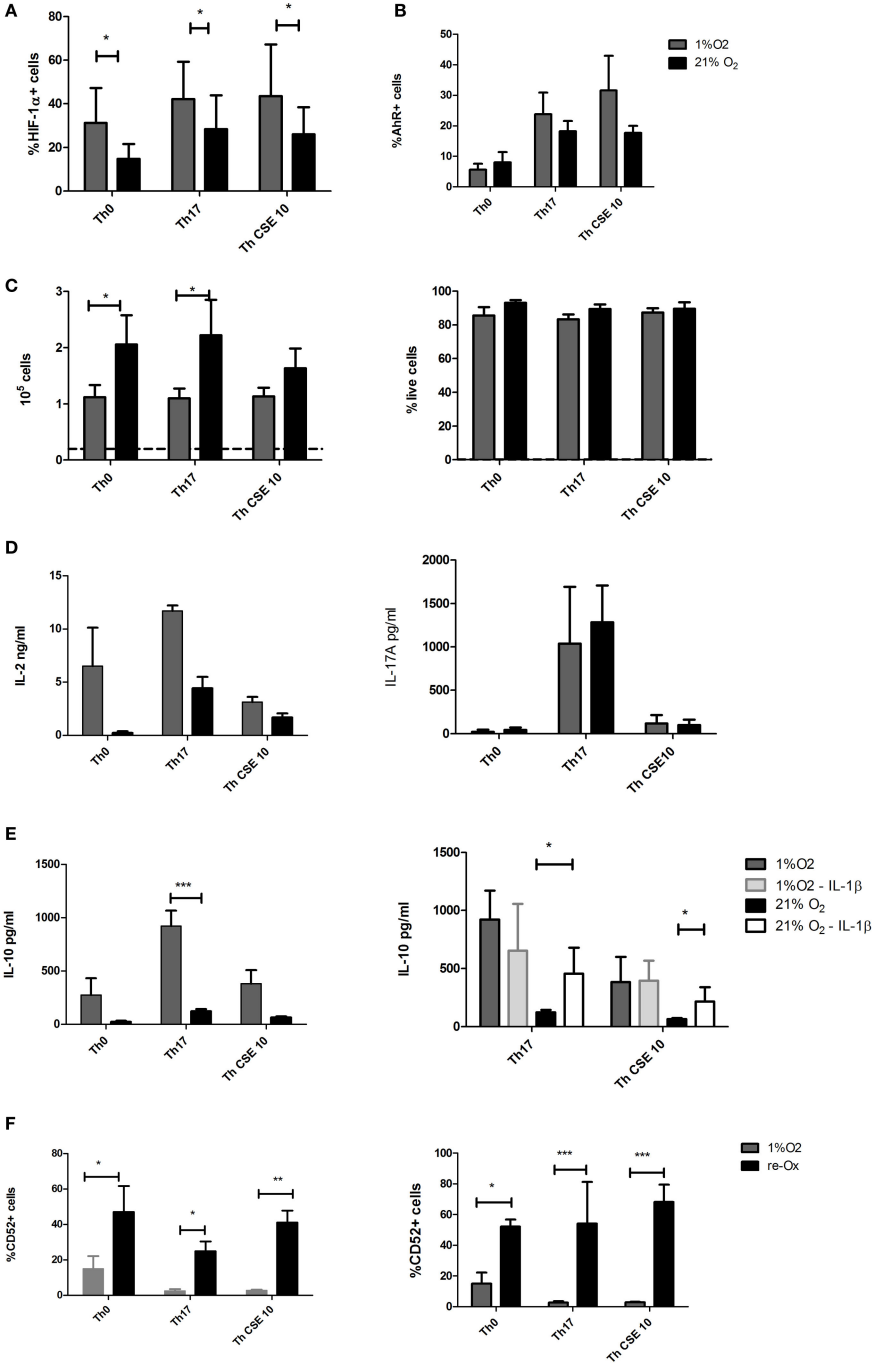
Hypoxia has complex effects on proliferation, cytokine production, and surface markers of T cells. T cells were polarized into Th17 under 1% O_2_ as well as in presence of cigarette smoke extract (CSE). **(A)** Hypoxia increased levels of HIF-1α in all T cell populations. **(B)** Aryl hydrocarbon receptor (AhR) levels showed slight insignificant increase as well. **(C)** Proliferation of T cells was reduced under low oxygen, without marked effect on the viability. The proliferation is showing absolute number of cells upon harvesting where the dashed line indicates starting amount of cells per study group. **(D)** Low oxygen treatment increased IL-2 production and unaltered IL-17A release by Th17 cells. **(E)** Th17 cells under hypoxia produced considerable amounts of IL-17A, and removal of IL-1β from Th17 polarization cocktail had little effect on IL-10 production at 1% O_2_ but increased IL-10 yield at 21% O_2_. **(F)** CD52 expression was reduced under low oxygen and restored after repeated exposure to 21% O_2_. The bars show mean + SD, *n* ≥ 3 in all experiments (*p < 0.05; **p < 0.005; ***p < 0.001).

Glucose-6-phosphate isomerase and ENO1 are genes transcriptionally activated by HIF-1α (25). To explore the role of HIF-1α on Th17 and Th CSE development, we performed polarization of T cells under hypoxia (1% O_2_), which is a strong physiological inducer of HIF-1α and such conditions are expected to mimic the oxygen conditions in the inflamed or peripheral tissue (8). In line with this, all T cell stimulated under hypoxia had significantly higher levels of HIF-1α compared to T cells stimulated under 21% O_2_ (Figure 3A). This was not the case for AhR levels (Figure 3B). Next, we determined the absolute proliferation and viability of T cell under low oxygen. The proliferation was significantly reduced for Th0 and Th17 cells, but not the Th CSE vs the ambient oxygen-cultured cells (Figure 3C, left panel). On average, Th0 and Th17 were proliferating approximately two times slower, while for Th CSE this coefficient was 1.44-fold. At the same time, we did not observe increased cell death (Figure 3C, right panel). Importantly, the IL-2 levels were increased under hypoxia (although insignificantly, Figure 3D, left panel), and IL-17A production was unaltered irrespective of increased HIF-1α levels. Strikingly, the rate of IL-10 production was increased by 1% O_2_ Th17 cells (Figure 3E, left panel). It has previously been shown that Th17 cells produce IL-10 in a pathogen-dependent manner and that this can be inhibited in the presence of IL-1β in the polarization cocktail (6). Therefore, we repeated the experiments without IL-1β. Indeed, the production of IL-10 was enhanced in absence of IL-1β but only for Th17 cells cultured at ambient oxygen (Figure 3E, right panel). Hence, it appears that IL-10 production by hypoxic Th17 cells was solely dependent on oxygen availability and that O_2_ determines the outcome of two distinct Th17 cell populations.

To characterize these T cells further, we performed cell surface marker staining, which showed that both Th17 cell populations have comparable levels of CD25, CD226, CD48, and CD38, while the levels of CD52 were significantly reduced by 1% O_2_ conditions (Figure 3F, left panel). CD52 or CAMPATH-1 is a 12 amino acid peptide anchored to glycosylphosphatidylinositol on the surface of mature lymphocytes and to some extent on monocytes and dendritic cells (DC) (26). CD52 has been shown to be released from cells with the help of the *PLCG1* gene product phospholipase C (26) to inhibit activation of other T-cells by impairing phosphorylation of the T-cell receptor-associated kinases Lck and Zap70. Our results may suggest that peripheral tolerance can be regulated in an oxygen-dependent manner involving CD52 expression. Indeed, when the T cells differentiated under hypoxia were reexposed to ambient oxygen concentrations for 48 h, the CD52 expression was significantly increased (Figure 3F, right panel).

### Hypoxic Environment Drives the Transcriptional Change in T Cells

**Figure 4:**
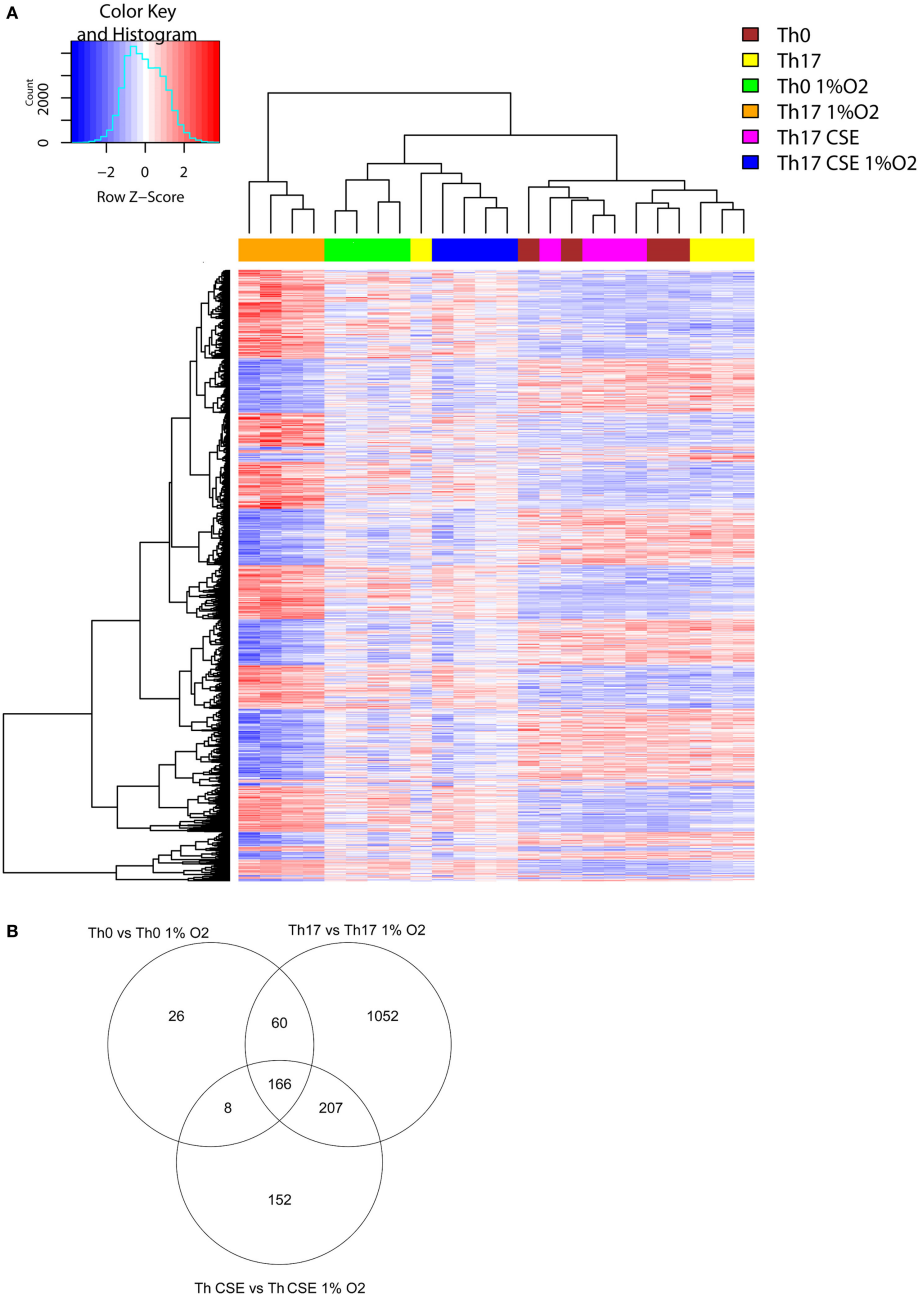
Transcription profiles of T cells. **(A)** Two-way hierarchical clustering of six T cell populations (*n* = 4) and 1,500 differentially expressed genes found in LIMMA analysis for the 1% O_2_ vs 21% O_2_. Clustering identifies differences between Th17 cells at 1% O_2_ and 21% O_2_. **(B)** Th17 cells had the most affected transcriptional profile after exposure to low oxygen in comparison to control and Th cigarette smoke extract (CSE) cells (Venn diagram).

To further explore the complex changes observed in T cells, we investigated the transcriptional changes upon exposure to a low oxygen environment and AhR ligand stimulation. Hierarchical clustering of 1,510 DEG separated the 1% O_2_ and 21% O_2_ cultured cells into two major mRNA expression groups. The two groups signified gene clusters specific for the Th17 cell population separating them from genes cluster groups specific for Th0 and Th17 CSE cell population (Figure 4A).

This showed that 166 DEGs (see Table S1 in Supplementary Material) were overlapping in all 3 subgroups due to change of oxygen environment, while Th0 had the least amount of specific DEGs (26, see Table S1 in Supplementary Material) and Th17 cells had the highest number of unique DEGs (1,052, see Table S1 in Supplementary Material) in comparison to cells conditioned under 21% O_2_ (Figure 4B, Venn diagram). All subgroups under 1% O_2_ showed upregulation of signal transducer and activator of transcription 3 (*STAT3)*, which is a key TF involved in differentiation of T cells and expression of, HIF-1α hydrolase Egl nine homolog 1 (*EGLN1*) that regulates the level of endogenous HIF-1α. We also observed downregulation of caspase 1 (*CASP1*), which is an enzyme involved in pro-inflammatory responses. In support of the observed reduction in cell surface levels of CD52, we also observed upregulation of *PLCG1*.

**Figure 5:**
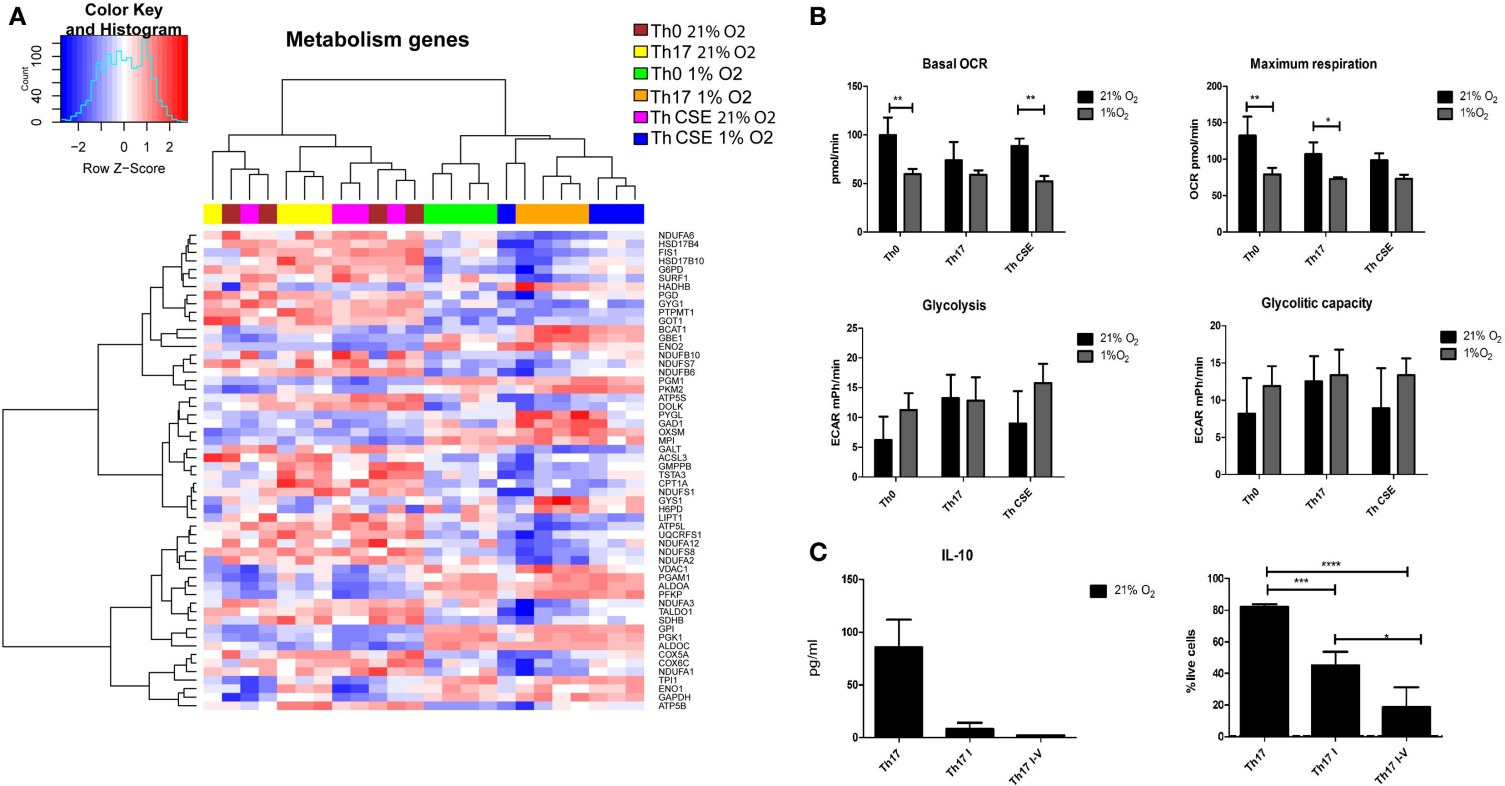
Metabolic changes in T cells depending on the polarization conditions. **(A)** Two-way hierarchical clustering of six T cell populations and genes involved in carbohydrate metabolism and the electron transport chain (ETC). 1% O_2_ T cells upregulate genes related to glycolysis and inhibit the ETC. **(B)** Hypoxia reduced oxygen consumption rate and maximum respiration with a tendency to increase glycolytic capacity of the cells, however, insignificant. **(C)** Chemical inhibition of ETC results in reduction of cell viability and does not lead to increased IL-10 production. Th17 I—rotenone treated; Th17 I–V—combined ETC inhibition. The bars show mean + SD, *n* = 3 (*p < 0.05; **p < 0.005; ***p < 0.001).

From Figures 2 and 4A, we observed that both CSE and low O_2_ influenced expression of genes involved in cell metabolism of nutrients including glucose. Figure 5 depicts that all 1% O_2_-cultured T cells upregulated several genes related to carbohydrate metabolism (*GPI, ENO2, PGK1, ALDOC, ALDOA, PFKP*, and *MPI*) and downregulated aspartate aminotransferase *GOT1* suggesting that the metabolism was switched into glycolysis, which is a well described feature of hypoxic cells (Figure 5A). However, short-term measurements did not show an increased glycolytic capacity on those cells while the OCR and respiration were reduced (Figure 5B). In line with the latter observation and as an important feature of the hypoxic cells, genes involved in oxidative phosphorylation (OxPhos) and ETC were downregulated. In fact, all parts of ETC were affected (Complexes I–V), and these features were particularly pronounced in the Th17 populations (Th17 and Th CSE). Furthermore, in Th17 cells at 1% O_2_ *PDHB*, which encodes beta subunit of mitochondrial pyruvate dehydrogenase (PDH), was downregulated. Downregulation of PDH is in line with previous reports suggesting that the Th17 phenotype supports glycolytic metabolism (27).

To investigate whether ETC inhibition under ambient oxygen condition would result in altered IL-10 production, we performed a series of experiments with selective inhibition of Complex I or combined inhibition of Complexes I, III, IV, and V. In contrast to ordinary Th17 polarization, chemical inhibition of ETC complexes reduced cytokine production and significantly reduced the viability of cells (Figure 5C).

### Adaptation of Th17 Cells to Low Oxygen Involves Complex Pathway Regulation

**Figure 6:**
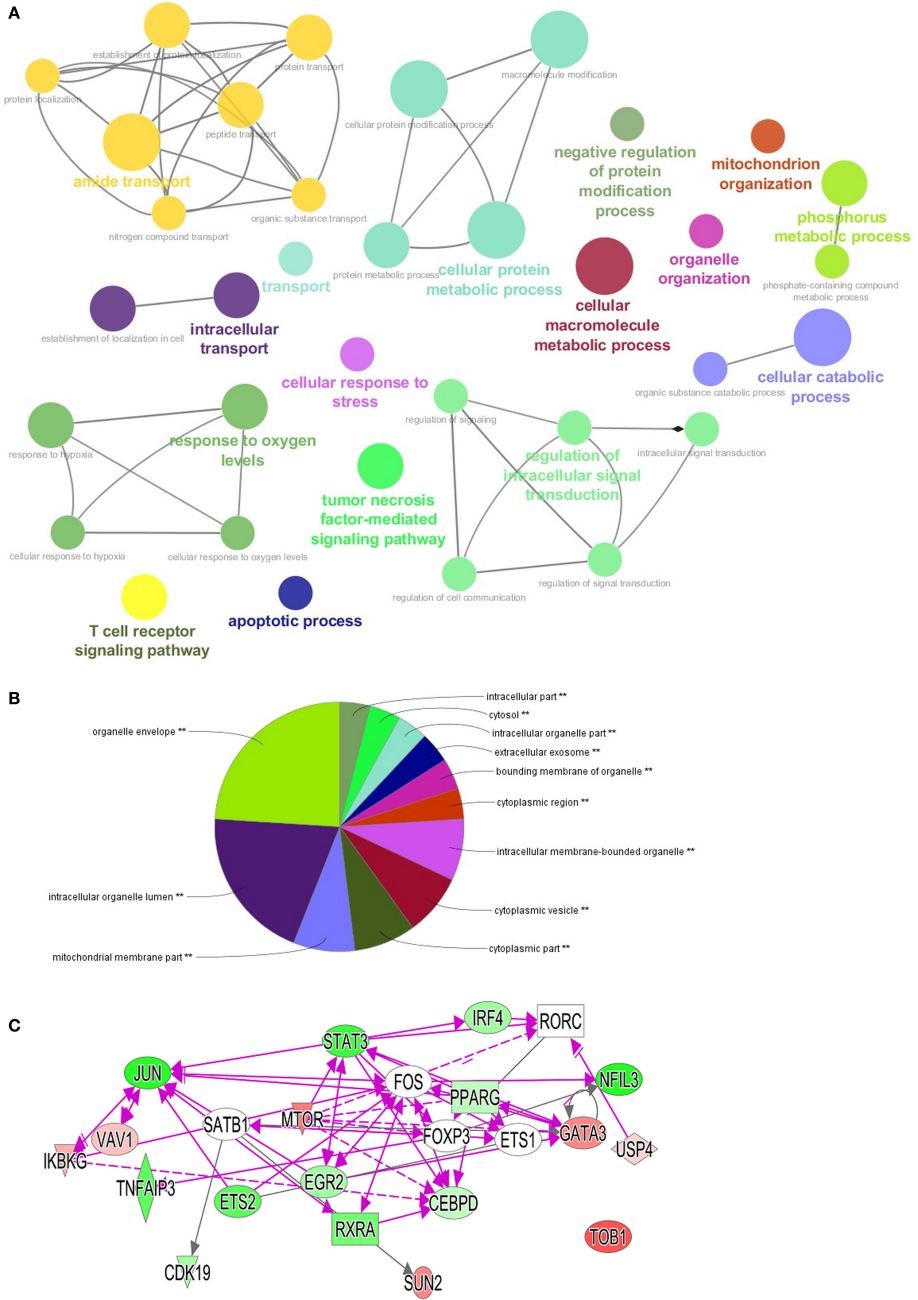
Visualization of pathways and processes affected in Th17 population at 1% O_2_. Differentially expressed genes (DEGs) with *p* values below 0.005 were used for pathway visualization using Gene Ontologies in ClueGo plug-in for Cytoscape software and IPA software (*n* = 1,385). **(A)** Top enriched biological processes shown as “nodes” using GO terms. Each network has a highlighted node (colored text) that summarizes the function of the network (automatically set by software), lines connect nodes within one network based on the Kappa score (14). **(B)** Visualization of DEGs localization using GO cellular component terms with ClueGo plug-in. **(C)** Transcriptional factors identified in the dataset green color of the node indicates upregulated at 1% O_2_, red—downregulated. Open elements—interaction partners not present among DEGs but involved in the networks. Triangle—kinase, rhomb—enzyme, oval—transcriptional factor, rectangle—nuclear receptor.

Our results demonstrated that the level of oxygen regulated major transcriptional changes in Th0 vs Th17 cells. Moreover, selective inhibition of only one involved element—ETC did not result in a phenotype observed at 1% O_2_. We therefore performed pathway analysis using the ClueGo plug-in for Cytoscape (14) and IPA software (28) to visualize complex changes under low oxygen. This revealed that 1% O_2_ induced major transcriptional change compared to conditions of 21% O_2_ that affected not only genes involved in response to hypoxia but also a plethora of other crucial processes in the Th17 cells—T cell differentiation, activation, TCR signaling, organelle organization, and intracellular transport (Figure 6A; Figure S2 in Supplementary Material). Protein metabolism and ubiquitination was significantly altered as well with 22 genes being downregulated and only 7 upregulated genes (see gene identity in Table S2 in Supplementary Material). Briefly, the expression of enzymes responsible for both mono- and poly-ubiquitination was reduced, while *BIRC3* (inhibitor of apoptosis), *USP12* (stabilization of TCR complex), and *DNAJB2* (protein that prevents RNA decay) were increased. We also observed that apoptosis-related genes, including *CASP10, CASP6, DFFB*, and *MAP3K5*, were downregulated, in line with our observation that cells under 1% O_2_ were not apoptotic. Similarly, genes responsible for cell cycle arrest such as protein phosphatase 2, catalytic subunit, and alpha and beta isozyme (PPP2CA and PPP2CB), the TFs E2F5 and E2F4 as well as 28 S-associated death-associated protein 3 were downregulated. Moreover, genes important for DNA repair such as poly (ADP ribose) polymerase family, member 9 (*PARP9*), histone deacetylase 3 (*HDAC3*), and *MAP2K* were upregulated in both Th17 and Th CSE cells. Importantly, upon analysis of cellular localization of involved genes, we found that the changes affect all compartments, with most of the changes related to organelles, and especially mitochondria (Figure 6B).

Th17 development is dependent on activation of specific signature TFs (29); however, TFs crucial for non-pathogenic Th17 cells are not well described. In our dataset, we detected already known TFs like *IRF4*, and others, less established for Th17 differentiation like *JUN, PPARG*, and *ETS2* (Figure 6C). Moreover, 1% O_2_ treatment downregulated *MTOR* in Th17 cells, a TF that is involved in polarization of *in vitro* generated Th17 cells (30), as well as *USP4*, a protease that promotes Th17 function under inflammatory conditions (31) and other key genes involved in AMPK, PI3K/AKT, and NF-κB pathways.

Lipid metabolism has been shown to be important for Th17 cells and their pathogenicity (32, 33). 1% O_2_ Th17 cells exhibited alterations in fatty acid β-oxidation and ketogenesis through upregulation of *HADHB* and downregulation of *ACSL3*. Interestingly, both Th17 and Th17 CSE cells have downregulated *SUCLG1*, enzyme that converts succinyl CoA to succinate, which are important signal in inflammation (34).

Unlike the ambient oxygen conditioned Th CSE, their 1% O_2_ counterparts showed a greater difference to Th0 cells and had upregulated *CYP1A1*, which is downstream target of AhR pathway and is involved in metabolism of various chemicals and toxins. Furthermore, Th CSE upregulated TFs *EGR1* and *JUND* and downregulated protein kinase A genes (*PRKACB)*, which are important regulators of T cell activation and differentiation (35, 36).

## Discussion

T helper (Th1, Th2, Th17, and Treg) cell plasticity is a known phenomenon when particular cells might switch their specificity depending on the surrounding cells and humoral conditions (37). The Th17 cells are currently subdivided into “pathogenic” and “non-pathogenic”; however, a major question is how this phenotype is balanced *in vivo*. Here, we report on how low oxygen environment that is commonly present in peripheral tissues, tumors, and hematopoietic organs might affect the development and polarization of T cell responses. We also show additional insights into the effects of CSE treatment on Th17 differentiation.

Cigarette smoke contains numerous non-contagious components that promote inflammation causing considerable morbidity and mortality by inducing cancer, chronic lung and vascular diseases, and oral disease (38). In addition to promotion of Th2 mediated immune reactions and suppression of Th1 function (39), CS is thought to reduce natural killer cell activity by preventing their capacity to produce INF-γ and TNF-α (40). Moreover, CS also influences DC function by altering their capability to produce prostaglandin-E2, IL-8, and IL-10, as well as suppressing the release of IL-12 and IL-23 (41). While CSE has been shown to increase expression of IL-17A and IL-17 receptor in the lung tissue (19) and certain individuals with chronic CS exposure have increased Th17 immunity (42), our data show that direct CSE exposure is not leading to Th17 polarization. This was shown by a reversed ability of Th CSE cells to produce IL-17 and IL-2. They were also not dividing in the same rate in comparison to Th0 or Th17 cells, despite that activation markers such as CD25 and CD226 were significantly upregulated compared to Th0 cells. Inhibition of Th17 polarization by CSE was further supported by our microarray analysis demonstrating similarity of the gene expression pattern of Th CSE to that of Th0 cells.

Cigarette smoke approaches the very first interface of the immune system—the mucosal surfaces lining the oral cavity, sinuses, and airways where ambient O_2_ is present (8). Several studies have reported that physiological or closer to physiological O_2_ conditions effect the proliferation, stimulation, and differentiation of T cells (8, 11, 43). Based on this and the fact that chemicals from CS are distributed to distant compartments with low O_2_, we tested the effects of CSE treatment on Th17 differentiation at physiological hypoxia (1% O_2_). Again, we observed that Th CSE cells were closely related to Th0 based on the microarray data even under hypoxia. We therefore postulate that CSE exposure does not promote Th17 polarization either at low or ambient oxygen conditions. This is further supported by inhibition of Th17 differentiation in a mouse model of EAE by AhR stimulation, which contrasted with *in vitro* studies (44). It should also be mentioned that the reactivity to CS and CSE might be a donor-specific issue as the HLA genotype is important for smoking-induced Th17 development in both humans and mice models of autoimmunity (17, 45). Hence, we cannot draw absolute conclusions based on our rather limited donor cohort.

In our experiments, we also observed that Th17 polarization under hypoxia produced cells with a very different gene expression repertoire compared to Th17 cells polarized under ambient oxygen. Strikingly, we found that Th17 polarization program leads to formation of IL-10-secreting Th17 cells, which might mimic the so-called non-pathogenic Th17 (5) or regulatory Th17 cells (46). The IL-10 production has been linked to IL-1β and specific pathogens earlier (6) and while our experiments support the IL-1β effects on Th17 cultures at ambient O_2_ conditions, the 1% O_2_ Th17 cells react in a different manner. It can be also speculated that the pathogen-dependent mechanisms would be affected by oxygen tension similar to observations in macrophages where inhibition of ETC by hypoxia impaired the antibacterial activity (47). In this study, we provide evidence that genes related to ETC are downregulated in Th17 cells (as well as other T cell populations) at 1%, which might be responsible for the conversion of pathogenic cells into non-pathogenic. In line with this, selective targeting of HIF-1α restored the activity of T cells regardless of tissue hypoxia (48). Importantly, we show that selective inhibition of Complex I by rotenone or combined treatment with other inhibitors of ETC complexes does not increase IL-10 production and reduces viability of the cells. Furthermore, these IL-10-producing Th17 cells support the concept of T cell plasticity as Th17 cells were shown to convert into a Treg like phenotype upon the resolution of inflammation and clearance from pathogen (49).

Hypoxia upregulated expression of phospholipase C in all studied T cell populations, which in turn is responsible for many processes including cleavage of CD52 from the membrane of CD52^high^ T cells (26). In line with this, we report reduction of CD52 levels under hypoxia and its restored expression after reoxygenation. The implication for this observation is not fully understood, but it can be speculated that compartmentalized oxygen levels can orchestrate differential immune reactions by the release of CD52 from circulating CD52^high^ T cells followed by binding to CD52^low^ cells in the periphery to prevent excessive inflammation. It might be that similar mechanism exists for other cell types, for example, DC as well; where it was shown that reoxygenation improved the DC maturation and function, improving priming of Th17 and Th1 cells (50).

As O_2_ levels are tissue-specific, differentiation of IL-10-producing Th17 cells are likely to be tissue-specifically regulated as well, a feature that may have important implications for local inflammatory responses. It has been demonstrated that non-pathogenic Th17 cells might be tissue-specific due to lipid metabolism and tissue-specific of CD5L (33). In our dataset, we found alterations of fatty acid metabolism genes *HADHB* and *ACLS3* in Th17 cells at 1% O_2_ but not in Th0 cells. Based on our results, we suggest that CD5L^+^ Th17 cells found in the gut mucosa might have specific lipid metabolism develop partly due to very low levels of oxygen present in this tissue (51). This might further function as a defense mechanism against both normal microflora as well as tolerance to food allergens. We suggest that in the absence of previously suggested pathogens and under hypoxia, TCR stimulation in presence of polarizing cytokines would lead to differentiation of cells into non-pathogenic subsets to prevent unnecessary immune reactions.

Deregulation of *HADHB* and *ACLS3* and downregulation of ETC complexes by O_2_ points to metabolic alterations as a mean of biologic characteristic of IL-10-producing Th17 cells induced by hypoxia. A common feature of cells with downregulated mitochondrial function is that they are glycolytic in nature (52). In fact, glycolytic activity has previously been shown to be of vital importance for Th17 cell functions and such cells are reported to have higher levels of expressed glycolytic genes in comparison to naïve T cells and Tregs. In line with this, we observed a series of genes important for glycolysis to be upregulated with one exception—*PDHB* that was downregulated. *PDHB* downregulation, however, is consistent with experimental evidence by others demonstrating that OxPhos is blocked in Th17 cells by inhibition of PDH activity through PDHK (27). PDHK is known to be upregulated by HIF-1α, while PDHB downregulation might be a specific feature of non-pathogenic IL-10-producing Th17 cells.

Our current study and observations by others (53) show that physiological hypoxia affects the transcriptional profile of T cells and Th17 cells. IRF4, RORC, BATF, STAT3, and several other factors have been long known as master regulators of Th17 lineage (29, 54). Non-pathogenic Th17 cells have been proposed to be dependent on c-MAF (7). In our study, the Th17 cells upregulated *c-JUN*, which supports the previously published observation of involvement of JUN, IRF4, and ETS in *IL10* transcription (55) and we also observed upregulation of PPARG, receptor that inhibits Th17 differentiation (56). Importantly, these studies were performed at ambient oxygen experimental conditions while in our settings these factors were upregulated by low oxygen tension that mimics the physiological concentration. Further, we also observed downregulation of USP4, which was previously shown as Th17 promoting protease under inflammation (31). We believe that it supports that idea that without specific pathogen the low oxygen environment would prevent excessive reaction of immune cells, in particular Th17.

In summary, we showed the complex effect of low oxygen conditions on the Th17 differentiation that resembles the so-called non-pathogenic Th17 cells, the origin of which is debated. We believe that knowledge of their differentiation and physiology is important for understanding of their role in different compartments of the body. Recent studies have provided a lot of essential data on Th17 development (57); however, we and others (58) believe that it is important to expand experimental settings to more physiological oxygen concentrations to reveal tissue physiology relevant questions taking into consideration new approaches and technologies (59).

## Accession Codes

The GEO accession number for the microarray data is GSE90882.

## Ethics Statement

This study was carried in accordance with the Norwegian laws and regulations with written informed consent from all subjects. All anonymous healthy blood donors gave written informed consent in accordance with the Declaration of Helsinki. Standard form was used by the Oslo Bloodbank (Ullevål hospital), and no additional approvals by ethical committee were required for the study.

## Author Contributions

RV and BS initiated the study and drafted the manuscript; RV and ZS conducted the experiments; RV, VN, and BS analyzed the results. All the authors reviewed the manuscript.

## Conflict of Interest Statement

The authors declare that the research was conducted in the absence of any commercial or financial relationships that could be construed as a potential conflict of interest.

## Supplementary Material

Figure S1 Intracellular staining for IL-17A was performed to see whether cigarette smoke extract (CSE) treatment blocked the release of cytokine. Experiment was performed without addition of Golgi block to either Th17 or Th CSE populations.

Figure S2 All pathways affected in Th17 population at 1% O_2_. Differentially expressed genes with *p* values below 0.005 were used for pathway analysis using IPA software (*n* = 1,385). Network interactions for canonical pathways with at least three shared genes are shown. Intensity of color indicates log *p* value.

Table S1 Summary of differentially expressed genes from the study.

Table S2 Pathways altered in Th17 cells under 1% oxygen.
