# Supplementary figures and images for: Th17 Polarization under Hypoxia Results in Increased IL-10 Production in a Pathogen-Independent Manner
Source: Front Immunol. 2017 Jun 19;8:698. doi: 10.3389/fimmu.2017.00698 (PMC5474482; doi:10.3389/fimmu.2017.00698)

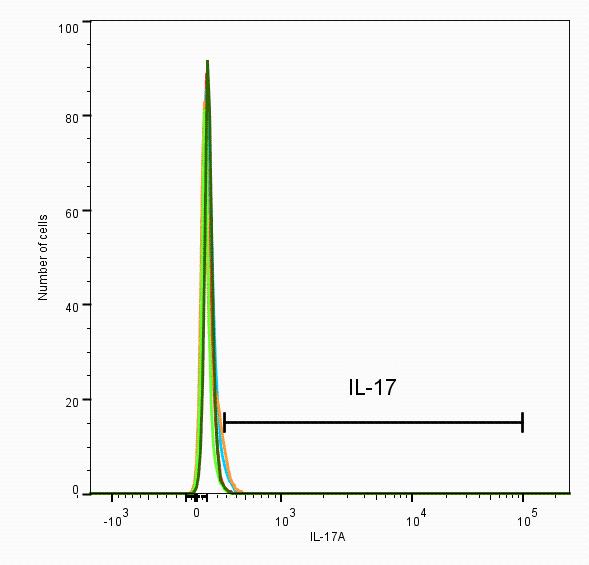

Supplement: Figure S1 — Intracellular staining for IL-17A was performed to see whether cigarette smoke extract (CSE) treatment blocked the release of cytokine. Experiment was performed without addition of Golgi block to either Th17 or Th CSE populations. [file Image_1.JPEG]

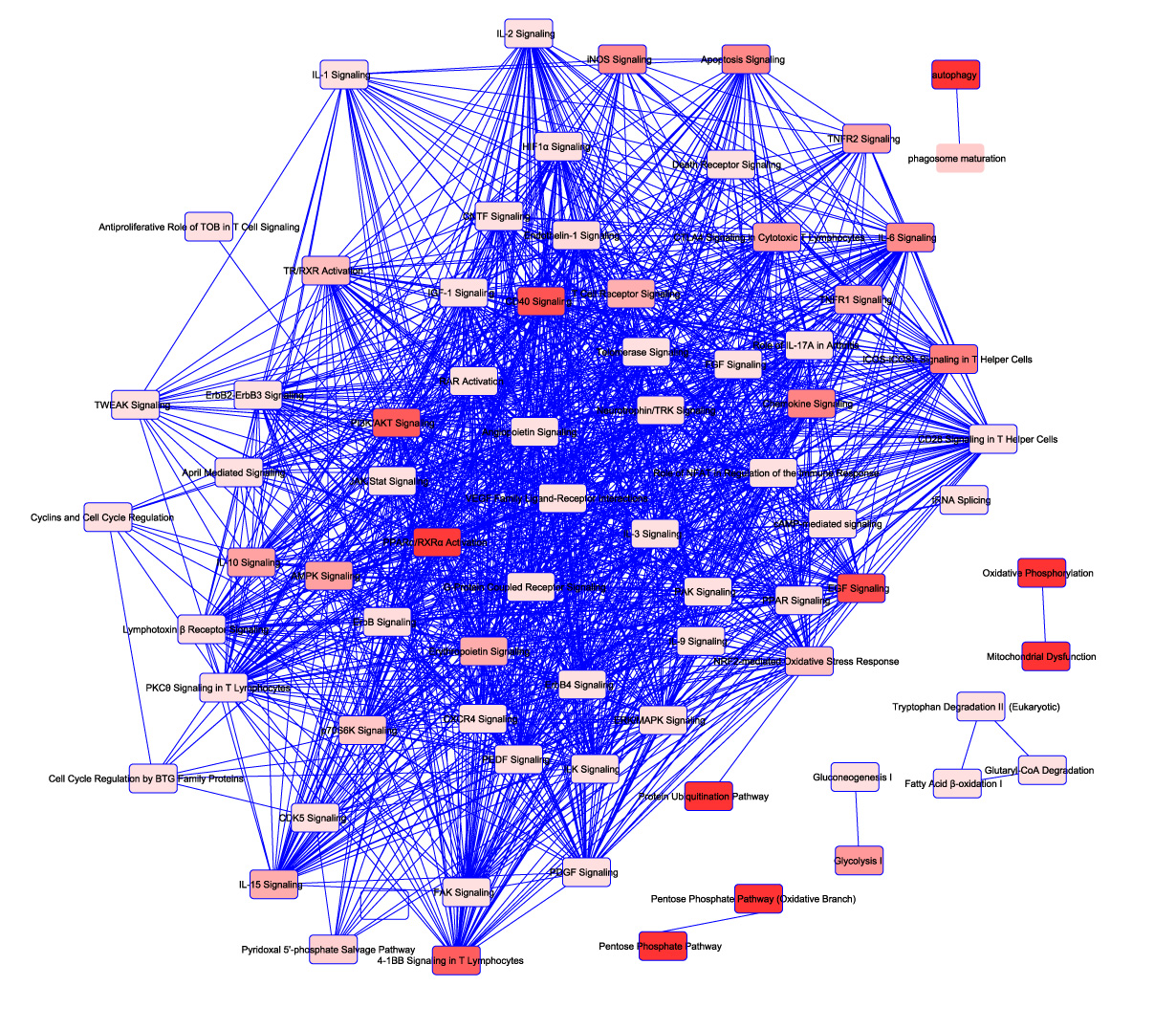

Supplement: Figure S2 — All pathways affected in Th17 population at 1% O2. Differentially expressed genes with p values below 0.005 were used for pathway analysis using IPA software (n = 1,385). Network interactions for canonical pathways with at least three shared genes are shown. Intensity of color indicates log p value. [file Image_2.TIF]
